# Cultural respect in midwifery service provision for Aboriginal women: longitudinal follow-up reveals the enduring legacy of targeted program initiatives

**DOI:** 10.1186/s12939-020-01325-x

**Published:** 2020-11-25

**Authors:** Rosalie D. Thackrah, Jennifer Wood, Sandra C. Thompson

**Affiliations:** 1 Western Australian Centre for Rural Health, University of Western Australia, Nedlands, Western Australia; 2 King Edward Memorial Hospital, Subiaco, Western Australia

**Keywords:** Aboriginal maternal health, Midwifery education, Transformative learning, Longitudinal follow-up, Cultural safety in service provision, Health disparities, Knowledge translation

## Abstract

**Background:**

Culturally competent maternity care provision to Aboriginal and Torres Strait Islander women was identified as a priority area by Australia’s National Maternity Services Plan in 2011. While midwifery programs responded by including core Indigenous content and community placements in curricula, little is known about whether knowledge learned, and insights gained in response to these initiatives have endured and been applied in clinical practice. This follow-up study explores the impact of a compulsory Indigenous unit and a remote clinical placement on two cohorts of non-Indigenous midwives who were participants in an earlier 2012–14 study.

**Methods:**

Fourteen non-Indigenous participants who were either students or recent graduates in 2012–14 were located and re-interviewed in 2019–20. In-depth interviews based on a semi-structured interview guide were conducted by telephone or face-to face; recordings were transcribed and thematically analysed using standard qualitative procedures.

**Results:**

Exposure to Indigenous content and settings during training had an enduring impact on participants’ midwifery practice; most felt better prepared to provide culturally safe care, build respectful relationships and advocate for improved services for Aboriginal women. Despite this positive legacy, they also expressed apprehension about causing offence and recognised their own knowledge deficits with regard to Aboriginal cultural practices. Organisational constraints, including restrictions on the number of family members accompanying a birthing mother were identified as barriers to optimal care; some positive organisational initiatives were also described.

**Conclusions:**

This follow-up study provides encouraging evidence that well-designed and delivered Indigenous content and community placement opportunities in midwifery programs can have a lasting impact on service provision to Aboriginal women, contribute to a more informed, empathetic and culturally competent maternity workforce and help catalyse health service changes towards more culturally safe care.

## Background

It has been nearly a decade since the publication of Australia’s National Maternity Services Plan (NMSP) which provided a framework to guide maternity policy and program development over a five-year period [1]. The Plan identified three priority areas with respect to Aboriginal and Torres Strait Islander^1^ women in recognition of their higher risk profile during pregnancy and birthing, and increased likelihood of delivering preterm and low birth weight babies. The priority areas include an expanded Aboriginal maternity workforce, increased cultural competence in the delivery of maternity care and development of dedicated Birthing on Country^2^ programs [2]. Despite some progress, it has been suggested that the NMSP implementation framework expired “. . . without notable results in these priority areas” [2]. Increased funding and commitment are required to train more Aboriginal midwives and ensure that birthing mothers deliver in culturally safe environments.

While the number of Aboriginal midwives is slowly increasing, there remains a huge shortfall to reach population parity [3]. Furthermore, “there has been no progress towards establishing and evaluating Birthing on Country services in remote and very remote Australia” although three projects are exploring this model in urban settings [2]. The introduction of compulsory Aboriginal content in midwifery programs and a *National Aboriginal and Torres Strait Islander Health Curriculum Framework*^3^ to guide implementation suggests some progress in the development of a culturally competent maternity workforce [4]. However, little is known about the translation into practice of knowledge acquired during training, measurement of cultural competency in maternity settings and whether there is a positive flow-on effect to Aboriginal women and their families from enhanced capabilities [2, 5, 6].

The focus of this paper is on the second priority of the NMSP, namely culturally competent maternity care provision for Aboriginal women, although the three priorities are interrelated. Early access to pregnancy care by Aboriginal women is influenced by a range of factors including widespread social disadvantage, proximity to services and lack of trust in the health care system due to past experiences and institutional racism; all act as disincentives to engage, with long-term consequences for maternal and infant health [2, 5–7]. Culturally safe practice is a requirement of the Australian Code of Conduct for Midwives; it is anticipated that program content and clinical placements will develop the cultural capabilities required to deliver safe care. Culturally safe practice includes: the incorporation of cross-cultural knowledge and historical factors into practice; respect for differences in cultural meanings and responses to health in maternity settings; recognition of the specific needs of Aboriginal and Torres Strait Islander women and their communities, and recognition and respect for customary law [1]. While the concept of cultural competence emphasises knowledge acquisition of health professionals, cultural safety focuses upon the recipients of care and the power differentials that exist in clinical encounters; significantly, it is only the recipients of care who can judge whether a health setting is culturally safe [8].

In the early 2000s, initiatives in medicine, nursing and midwifery commenced in response to the Committee of Deans of Australasian Medical Schools (CDAMS) *Indigenous Health Curriculum Framework* and the Indigenous Nursing Education Working Group report *‘gettin em n keepin em*’ [9, 10]. Embedding Indigenous content into curricula was given a further boost by the seminal project *Indigenous Cultural Competency in Australian Universities*. Undertaken by Universities Australia and the Indigenous Higher Education Advisory Council (2009–11), it developed a National Best Practice Framework, which had as a key component, the Indigenisation of all university curricula [11]. Informed, culturally competent graduates were considered essential to efforts to reduce socio-economic and health disparities experienced by Aboriginal Australians; medical and health sciences accreditation bodies responded by mandating compulsory Aboriginal content in programs. Numerous initiatives across the country have provided evidence of a range of transformative impacts on students from classroom content and cultural immersion experiences [12–17]. Despite some encouraging findings, doubt has been expressed about a link between educational initiatives and improved outcomes for Aboriginal people due, in part, to embedded culture within organisations and barriers with implementation of knowledge [18–20]. Furthermore, few longitudinal studies have been conducted to determine whether the knowledge acquired, and any attitude change observed in students is maintained over time and translated to clinical practice.

A number of theories have been posited to explain the experience of learning about Aboriginal history and cultures, the shift in attitudes that can occur and the positive impact of cultural immersion experiences. Mills and Creedy usefully employ a ‘pedagogy of discomfort’ framework to explore non-Indigenous students’ emotional responses to content and the process of critical reflection [13]. Despite discomfort, students can move from ‘spectating’ to ‘witnessing’; the former being a passive, comfort-zone position which maintains distance and absolves one of responsibility, while the latter involves a deeper engagement and includes an ethical and active dimension that can lead to transformational changes [13]. These changes can also be understood in the context of transformational learning theory [12, 21]. Bullen’s large quantitative study which explored students’ attitude change and preparedness to work in Aboriginal contexts upon completion of an Indigenous unit identified small but significant shifts and attributed these in part to the role of critical self-reflection, an important component of transformational learning [12]. In addition, experiential and ‘situated learning’ frameworks are frequently employed to understand the positive impact of culturally immersive experiences [22]. Service-learning opportunities where students learn from and contribute to communities can encourage socially responsive behaviours, dispel stereotypes and improve satisfaction with the learning experience [22].

This qualitative study examines the enduring impact of Indigenous content and remote clinical placements on 14 non-Indigenous midwives who have been practising for up to seven years. During their training, or in several cases immediately upon completion, participants took part in classroom observations, pre and post-unit surveys, and/or interviews in 2012–14 for a larger doctoral study. Participants had completed either a new Indigenous unit in the first year of their program or a remote clinical placement in their final year.

The unit formed part of an interprofessional common first year introduced for health science students at a Western Australian university in 2012. It was designed with substantial Aboriginal input and taught by a team of Aboriginal and non-Aboriginal staff. Its pedagogical framework was informed by the concepts of cultural safety with its focus on the recipients of care and cultural security, which stresses the incorporation of Aboriginal cultural values in health service delivery [6]. Vodcasts developed by the unit’s Aboriginal co-ordinator featured informal conversations (yarning) with Aboriginal academics and community members [6]. Topics ranged from diversity within communities, family structures, past policies and practices, and cultural beliefs surrounding health, pregnancy and birthing; personal stories highlighting the impact of dispossession, cultural loss, removal from families, resilience and language revitalisation were also included [6, 15]. The unit received a national award for excellence in 2016.

The remote Ngaanyatjarra Lands clinical placement became available to final year students in 2010 and as such pre-dated the new unit, although students had received some instruction in Aboriginal health. Those who expressed interest and were selected had an opportunity to undertake the 1–2 week placement, which was supervised by the Aboriginal Community Controlled Health Service midwife on the Lands [6, 16]. The aim of the placement was to provide exposure to health service delivery in isolated, traditional Aboriginal communities and provide supervised health care to women. Students observed the challenges of service delivery in remote settings and the difficulties encountered by women who had to re-locate for birthing; they developed communication skills in sensitive ‘women’s business’^4^ and participated in culturally appropriate health promotion activities designed with and for community members [16, 23].

A series of publications have provided a detailed analysis of these program initiatives [6, 15, 16, 23]. Conclusions highlighted the capacity for attitude change in response to well-designed content delivered in a safe setting and the positive impact of remote placements on student learning, despite their short duration. The aim of this study was to determine whether the attitude change observed, and the impact of the placement was sustained over time and contributed to a more informed, culturally respectful workforce cognisant of Aboriginal women’s needs. Participants in this follow-up study were located and re-interviewed in 2019–20 to investigate their clinical experiences and the legacy of these program initiatives.

## Methods

Follow-up studies where original participants are located after a period of time and re-interviewed are one of a number of qualitative longitudinal research methodologies [24]. In this study, data was gathered cross-sectionally at two periods of time: initially upon completion of a unit of study or a clinical placement and subsequently after participants spent time in the workforce. Longitudinal designs are increasingly used in health services research to explore patients’ experiences and the impact of clinical interventions [24]. On this occasion, the impact of earlier educational interventions on midwives’ provision of care to Aboriginal women was under investigation.

This study is based upon follow-up of two different although related primary cohorts of students. The first was a class of 15 direct-entry, first year midwifery students who undertook a new, compulsory Indigenous Health and Cultures unit at a Western Australian university in 2012. In 2019, nine of the 15 participants were practising midwives, the criterion for interview and inclusion in the current study. Four of the original students had not completed the midwifery program and a further two completed but did not practise. Of the nine midwives eligible to participate, seven were interviewed, one was not interested, and one could not be located. The second group comprised those who completed a final year clinical placement on the remote Ngaanyatjarra Lands in Western Australia in 2010–13. All seven of the original participants were located and re-interviewed; each was a practising midwife.

Past participants were located using the extensive networks of one of the authors (JW), a clinical midwife consultant at a major teaching hospital in Perth and former co-ordinator of the midwifery program that participants had completed. Potential participants were informed about the study and invited to contact the lead researcher (RDT) if interested and willing to be interviewed. When contact was made, the lead researcher provided Interviewees with a Participant Information and Consent Form; consent was given either by the return of a signed form or formalised verbally at the commencement of a recorded interview. The high response rate from the two groups (7/9 and 7/7) is attributed to their earlier participation in the study and rapport already established between the lead researcher, the clinical coordinator and the midwives. To avoid potential bias arising from her former role and professional association with participants, JW did not conduct any interviews.

The lead researcher (RDT) conducted all semi-structured interviews; most were completed by telephone (12) and two were face-to-face, one in the researcher’s home and one in an interviewee’s home. An interview schedule developed by the research team guided the direction of the interview, however, individual circumstances occasionally resulted in the addition of further questions. Interviews lasted between 30 and 75 min, the majority being more extensive. Each interview was recorded with consent and transcribed by the lead author. Original audio files and transcripts were secured in the lead researcher’s password-protected computer. Transcripts, which were shared within the research team, were subsequently coded for emerging themes. Standard qualitative techniques were employed including immersion in the data through the transcription process, annotation and coding arising from multiple readings, bracketing of key quotations and categorisation and refinement of emerging themes [25]. Transformative and situated learning theories provided frames of reference for interpretation of the findings, with a particular focus on the enduring impact of program learning. Approval for the study was granted by the Human Research Ethics Office at the University of Western Australia in 2018.

## Results

### Demographic and employment characteristics of participants

All participants (14) were female, and their ages ranged from late 20s to mid 40s. The majority had partners and children; two were on maternity leave at the time of interview. Ten participants practised in the Perth metropolitan area, two in regional/rural areas (Bunbury and Katanning), and one in Brisbane. One participant had recently moved to Uganda as a volunteer and another, currently working in Perth, had volunteered in Mozambique. With only one exception, all midwives worked in the public health system, although one combined this with private practice; another was employed by Boodjari Yorgas in Katanning, part of the Great Southern Aboriginal Health Service. One participant had been keen to work in the Kimberley upon graduation but was not accepted due to her direct-entry midwifery qualification. A number expressed concern that the lack of a nursing qualification limited their opportunities for rural employment upon graduation.

At the time of interview, half of the participants (7) were working where they had completed their graduate program, the other half had held numerous positions. Reasons for staying included work satisfaction, convenience of location and being risk averse due to marriage breakdown, while those who moved cited partners’ employment, travel, fresh opportunities and passion to work in a developing country.

Involvement in a Midwifery Group Practice,^5^ which allows for relationship-building with women, was the most frequently cited positive aspect of participants’ work. Other positive aspects included working with vulnerable women and those from diverse cultures, autonomy and organisational support offered to staff, and opportunities to preceptor midwifery students. Most participants made reference to the rewards derived from developing rapport with women. For one midwife who provided antenatal care specifically to Aboriginal women, connecting with families was also rewarding. *“I enjoy the grandmothers and the aunties coming together when a baby is born and home visits when the aunties are there listening to what I tell her. . . I love the fact that there’s a safety net around her”.* The majority identified collegial support and debriefing as sources of strength on difficult days and powerful antidotes to stress. For others, time alone was required: *“we talk about going home and “washing off the day” . . . I always go for a walk down by the creek with the dogs, I can’t talk for about 20 minutes, I must get outside and then I’m okay”.*

Less positive aspects of their work included: shift work and being on call (especially for those with young children), the dominance of doctors in some settings, high levels of stress and heavy workloads, and lack of opportunities to birth Aboriginal women at a low risk Family Birthing Centre.

### Interactions with Aboriginal women and their families: the role of unit content in building respectful relationships

The six participants employed in public hospitals had opportunities to work with Aboriginal patients, although the extent of involvement varied. Several had also worked with Aboriginal midwives, but Aboriginal Liaison Officers and Health Workers were more often colleagues due to their greater presence. All participants considered that knowledge acquired in the Indigenous Health and Cultures unit informed their practice and many cited examples of how they continue to apply learnings. Awareness of systemic disadvantage, a greater capacity for empathy, being less judgemental, recognising and responding to vulnerability, and an appreciation of the relationship between culture and health were all attributed to the unit content.

It was generally recognised that empathy arises out of a connection with others; it is associated with a capacity to understand or feel what another person is experiencing from within their frame of reference. For these participants, empathy arose from an understanding of colonial history and its ongoing impact. For many, this was eye-opening as it had not been encountered before. *“It helped by learning about history, what Aboriginal people have been through, you need to understand people’s history to develop empathy”.* It was also understood that empathy is pivotal to improved communication and rapport with Aboriginal women. One participant described how she defused a potentially volatile situation by establishing a connection with a distressed, recently delivered mother. “*She told me to go away in not a very nice way, but I ended up building rapport with her, and I think it was the training I had that helped me do that.”*

Several participants made reference to the ‘ripple effect’ and described how knowledge gained was discussed in social situations and with family members, especially children. The transferability of content to culturally diverse settings was also raised by those who worked with migrant and refugee populations. *“It opened your eyes to other cultures too.*. *. [this was] an unanticipated outcome but opened my mind to the connection between health and culture in general”.*

Despite acknowledging the value of the unit, most participants identified gaps in the content and remained apprehensive when working with Aboriginal women, worried about causing offence.

*“I still feel quite tentative and nervous about engaging with Indigenous clients and I think that’s quite common. There is a concern that we are going to do something wrong, offend someone. I think Aboriginal culture is still quite mysterious to us and that’s a gap in our knowledge. It speaks to ignorance, which I don’t think Indigenous people are perpetuating.. . they’ll often say, ‘if you are not sure just ask’.. . so we’ve got a long way to go”.*

*“I’m still somewhat apprehensive about working with Aboriginal women although I want to, because I don’t want to offend them. I don’t understand the culture well enough to know what is offensive or not, we did learn some of that in class (but) I think it comes with exposure”.*

### Interactions with Aboriginal women and their families: the role of remote clinical placements in building respectful relationships

All participants who completed the remote Ngaanyatjarra Lands placement had worked with pregnant and birthing Aboriginal women and most had a heightened awareness of unmet needs and organisational limitations with respect to cultural safety and security. While they had prior exposure to some Aboriginal content in their program, it was the culturally immersive placement experience that reinforced that knowledge and had the most impact on their practice. On re-interview, issues around communication, cultural protocols and the remote service delivery were identified as powerful learnings acquired; learnings they continued to apply on a regular basis. When asked about the importance of the remote placement and the application of learnings to practice, one participant who had just taken on a preceptor role commented:

*“It was hugely important. I think it made me a better clinician. .. I can tease information out of women because I have a better understanding, much better than if I hadn’t gone. Now that I’m teaching students, that experience will hopefully enrich their learning as well.*

*My practice is richer because of it but I’m also richer because of it. . .”*

Others commented:

*“I feel so fortunate to have had that experience. It will stay with me forever and I use what I learned in every encounter with Aboriginal women”.*

*“I’ll never forget that experience, never. I won’t forget how isolated it was, how totally alien it would be for these women if they came to Perth, navigating the system, and the language barrier and cultural differences. I’m acutely aware of these things now. Content in the program was important too, it was a history lesson for me, to understand the disparities and the effects of that and then going to the Lands really cemented it all, it consolidated what I learned”.*

All participants recognised that midwifery practice must respond to Aboriginal women’s needs and that midwives should be aware of power differentials, body language, communication patterns, shyness associated with ‘women’s business’ and behaviour bearing the hallmarks of implicit racism. One noted that while she highly valued the remote experience, she found that it had changed her in ways that could be socially awkward.

*“That experience was profound, but it separates you, it makes you different. Sometimes I feel like an outsider. .. It can be difficult socially when you realise how differently people think, some get it and others simply don’t.”*

Reference was also made to mistakes that occurred despite the best of intentions

*“I still make mistakes. I mentioned that I went to school with kids from Roelands Mission in a FASD (Foetal Alcohol Spectrum Disorder) meeting and an Aboriginal man immediately put up a barrier.. . now I understand that there were allegations of sexual abuse.. . I thought it would be a connecting factor, but it put me on the other side, even when you are treading carefully, you can get it wrong. There was a closed door. .. he must have had some bad experiences and knew much more than me.”*

All participants highlighted how their experiences in remote settings provided opportunities to connect with women and make them feel more comfortable in a city environment.

*“I understand how overwhelming it would be to come to Perth, how daunting, I think I’m kinder because of that knowledge, I think I try to reduce fear and awkwardness because of that experience. In general, the application is more in the area of social skills and communication that clinical skills.”*

These participants also identified numerous gaps in their knowledge including cultural diversity between language groups, limited awareness of the relationships between people, and inadequate knowledge of Aboriginal women’s health in terms of lifestyle and co-morbidities. For some, these gaps were filled by ‘learning on the job’. Others were less sure of their effectiveness. *“I think you’d have to ask the women I care for, I’m not always able to identify something they need and most of the women wouldn’t give me that feedback. I think I’m doing well but they are the best judge of that.”*

### Health professionals’ perceptions of Aboriginal women’s health service experiences and organisational efforts to improve service delivery

Participants from both groups were asked about Aboriginal women’s perceptions of maternity service provision. While clearly unable to speak on their behalf, those who commented drew from their observations and conversations with women in a variety of settings. The most frequently cited issues related to limitations on the number of support people allowed and the isolation felt by women from country areas.

*“I remember a young 17-year-old; she was all alone.. . she rang some family members who couldn’t get in, I really felt for her. Her experience wasn’t so great, but there seemed nothing the health service could do to change that.”*

Several participants described how relatives of country patients slept in cars near regional and city hospitals because they were not permitted to be with a birthing mother. It was also suggested that the experience of women can be influenced by the need to leave her community to birth.

*“If they [Aboriginal women] live close to Perth, I think the experience is very different from someone from the Kimberley for example. It’s freezing down here in winter, little social support. Local women are used to coming to clinic. Women from the north look scared and withdrawn.. .”*

*“There was an Aboriginal woman from Derby, and she was here for weeks and weeks. She was so lonely and isolated. I became quite close to her.. . I was there for the caesarean and while not many words were spoken between us, it made me realise the huge upheaval being away from her family. I bought her some baby outfits and when she was leaving, she had the baby in one of them.. . so much needs to change so it’s not such an alienating, isolating experience”.*

Numerous organisational efforts to improve service delivery to Aboriginal women were described by participants. One who worked in a low-risk Family Birthing Centre (FBC) at a major teaching hospital was committed to forging stronger links with Aboriginal maternity groups at other hospitals and disseminating information about the FBC. *“Last year nine Aboriginal women went through to deliver. We are renovating the birthing rooms and inviting Aboriginal women to have some input, to make it more welcoming..*. *low risk births can be a problem for Aboriginal women... but we are spreading the word”.* Another commented that ideas from participants at a recent Aboriginal women’s birthing conference were incorporated into the design of the new room and that feedback had been positive.

*“We aim to get more low risk Aboriginal woman through... recently a young woman who had a beautiful birth in that room came back for a morning tea to talk about her experience. She felt this was the closest she could get to birthing on country. .. she could have any number of people to support her”.*

Participants also spoke of the important role of Aboriginal Liaison Officers and the support they provide to women, especially in the nursery where *“they can sit down with new mums and explain things”.* It was suggested, however, that their numbers appear to have declined. *“I used to see them a lot but can’t say I see them as often. .. other midwives have spoken about a drop on the numbers of ALOs”.*

## Discussion

The participants in this study were an extension of an earlier research project conducted in 2012–2014. At the time, those who completed the Indigenous Health and Cultures unit were found to be largely responsive to the content, although pockets of resistance and emotional discomfort were identified in some of those enrolled in the compulsory unit. Evidence of increased knowledge, positive attitude change and improved critical thinking skills arose from pre and post-unit surveys, classroom observations and interviews. Unresolved issues around race and what constitutes racist behaviour were also discerned [15]. Those who undertook the remote placement on the Ngaanyatjarra Lands reported valuable insights gained around the impact of isolation on health service delivery to Aboriginal women, the strength of cultural traditions, especially surrounding pregnancy and birthing and the importance of communication in the context of sensitive ‘women’s business’ [16, 23].

The follow-up interviews conducted in 2019–20 explored the longer-term impact of program initiatives to better prepare midwives to work with Aboriginal women and their families. Three themes emerged: empathy and relationship-building; apprehension and anxieties and organisational initiatives and constraints. They highlight the powerful and enduring nature of student learning on longer-term midwifery practice but also reinforce the fragility of relationship-building in settings where Aboriginal safety and security is not prioritised.

### Empathy and relationship-building

Midwives described the various ways in which they had been changed by learning about the impact of colonisation on Aboriginal people and the longer-term socio-economic consequences of past policies and practices; most had limited knowledge of this history and experienced discomfort when learning about past and present injustices. Most recognised this knowledge as essential to establishing rapport with Aboriginal women and required an awareness of their own privileged position in society. Critical self-reflection involving a willingness to question deeply held assumptions and consideration of the views and perspectives of others can be precursors to shifts in attitudes according to Mezirow’s transformational learning theory [12, 21]. Mills and Creedy noted however, that “research that measures learning in this space” is not widespread and much remains unknown about the emotional responses of both students and staff with uncomfortable content [13]. Furthermore, Isaacs and colleagues, who explored the concept of ‘cultural desire’ among nursing students upon completion of an Indigenous health unit, found no evidence of an increased desire to practise in a culturally competent manner; they noted that enhanced knowledge and skills “might all be in vain if there is no desire to be culturally competent” [26]. Thus, it cannot be assumed that simply exposing students to content during their training will translate into more empathetic professionals who deliver superior care.

While few studies have explored the legacy of program initiatives designed to improve cultural capabilities in midwifery practice, Brown and colleagues’ study of midwives providing care to Aboriginal women highlighted their awareness of the importance of communication with women, which was facilitated by valuing family members, support people and building networks with Aboriginal health care providers [27]. However, the authors reported midwives’ limited understanding of the concept of cultural safety and of “the power they were afforded as caregivers, there was no clear respect for difference or even an understanding of their own cultures and the impact that might have on the women they cared for” [27]. It was noted that while all the midwives had completed mandatory cultural awareness training, most had qualified more than 25 years ago, before cultural safety principles were embedded in midwifery curricula.

The participants in this study identified empathy, the ability to understand and share the feelings of another, as a capability acquired through exposure to Indigenous content in their program and/or from ‘in situ’ learning on a remote clinical placement. Effective communication arises out of empathy and is essential to relationship-building. That the unit was successful in this regard might be attributed in part to the strong presence of Aboriginal voices in the classroom using vodcasts as a means of disseminating personal stories [15]. These were observed to have a powerful impact at the time and were alluded to by some participants on re-interview. Those who had a remote experience benefited from increased exposure which provided a context for content learned in the classroom and likely the discomfiture of being in a place and space that is unfamiliar. They often recalled how they applied learnings about communication styles; examples cited revealed improved interactions with women who felt alienated and alone in maternity care settings, especially those from rural and remote areas. Participants from both cohorts, however, were conscious of making mistakes and concerned about the potential for causing offence.

### Apprehension and anxieties

Despite feeling better prepared to work with Aboriginal women and their families than many of their colleagues, most participants were aware of their shortfalls when it came to providing culturally safe care. Unlike the midwives surveyed by Brown and colleagues, these participants were largely cognisant of culturally safe principles, but not always confident they were putting them into practice. Most recognised that Aboriginal women themselves were the ultimate arbiters of culturally safe practice. These findings are suggestive of cultural humility, the concept explored by Tervalon and Murray-Garcia [28], which requires practitioners to engage in self-reflection, be alert to power imbalances that exist in clinical encounters and to develop respectful partnerships. Humility embraces the notion that a patient’s knowledge about themselves and their own experiences are important parts of the therapeutic process; humility is seen as a professional strength that leads to culturally inclusive care [28].

Apprehension associated with providing care to Aboriginal women and anxieties about causing offence are likely borne out of increased knowledge that leads to an awareness of knowledge deficits: “the more I learned the more I realised how little I know”. A number of participants recalled mistakes inadvertently made and the sense of accompanying shame, which suggests a strong self-awareness, recognition of the importance of building relationships in their caregiving, and a willingness to learn. Many gaps in knowledge were identified, including the extent of cultural diversity between language groups, the nature and significance of extended kin relationships and the extent of co-morbidities that can impact optimal birth outcomes. Some actively sought to address these knowledge deficits by establishing ties with Aboriginal health organisations, attending Aboriginal maternity conferences and through self-education. It is noteworthy that even the midwife who had worked in an Aboriginal community-controlled organisation for several years acknowledged that her learning had only just begun and the women were her teachers.

### Organisational initiatives and constraints

While Indigenous content in midwifery programs and opportunities for rural and remote placements can contribute to enhanced cultural capabilities, midwives can experience organisational barriers when applying cultural learnings to practice. The *Birthing on Noongar Boodjar*^6^ study, which gathered evidence from Aboriginal birthing mothers about their maternity experiences highlighted unsatisfactory and discriminatory care and the urgent need to “improve the cultural competence of non-Aboriginal people providing care to pregnant Aboriginal women” [5]. The importance of “cultural learning opportunities” provided by Aboriginal staff was noted, “particularly in relation to the centrality of family being present during childbirth” [5]. The role of non-Aboriginal “cultural champions” who demonstrated cultural security and advocated for culturally respectful maternity provision was also identified as a best practice model.

A number of participants in this study had in fact assumed the role of de facto “cultural champion” and actively advocated for improved service provision for Aboriginal women. Involvement of Aboriginal women in potential initiatives was imperative; their advice was sought and incorporated into improvements including a new, culturally respectful birthing room at a large public hospital. Despite some successes, participants recognised some of the barriers to creating change: inadequate staff training, disinterest and disrespect of some staff, under-representation of Aboriginal health professionals and inflexible organisational structures. System inflexibility was particularly evident when the centrality of the family in the birthing process was not recognised or accommodated. Marriott and colleagues noted with reference to the *Birthing on Noongar Boodjar* findings:

*“Birthing, Senior and Elder Women all referred to health service restrictions on the number of support people allowed in a birth (labour) suite or in postnatal rooms, and midwives not always understanding the strength and importance of kinship relationships, or the custom of childbirth as a community event. The lack of flexibility, in combination with the absence of continuity of carer or woman centred care. .. were all considered to have negative impacts, leading to women feeling unsafe and fearful”* [7]*.*

Most participants in this study highly valued their learning experiences related to Indigenous program content; they felt better equipped than many of their colleagues to provide culturally safe care, despite anxieties about causing offence. While not asked directly whether their attitudes and practices had changed since they commenced midwifery practice, a number alluded to increased confidence that arises from experience and a heightened awareness of inappropriate behaviour among some colleagues. Participants were cognisant of cultural safety principles; most applied them in practice and four were actively involved in advocacy. Interest and engagement in advocacy likely arose from professional experiences, commitment and opportunities to apply learnings as it was not included in the training program. Most participants also were able to identify knowledge deficits regarding Aboriginal cultural practices which, as new graduates they could not articulate.

It is noteworthy that several differences were observed between the two cohorts of participants. The majority who completed the first-year unit were exposed to Aboriginal history and culture for the first time; it was unsettling but also had an immediate and profound impact. Despite gaps in their knowledge, most appeared to apply what they had learned about Aboriginal family structures, the importance of kin and the legacy of past policies and practices on health-seeking behaviour in their interactions with Aboriginal women. Of course, there were exceptions, as an earlier example of poor practice reveals. On the other hand, the final year students undertook their placement with some prior knowledge of the social determinants of Aboriginal health. The most powerful impact of this experience arose from exposure to isolated, socially disadvantaged but culturally rich traditional Aboriginal communities. Later, as early career midwives, many cited knowledge of communication patterns, cultural protocols, and awareness of the cultural dislocation and loneliness experienced by Aboriginal women in city hospitals as important legacies of the placement. The culturally immersive experience allowed participants to consolidate prior knowledge and contextualise their learning; it enhanced confidence in subsequent clinical practice and also alerted them to organisational failures.

These findings provide encouraging evidence that well-designed and delivered Indigenous content in midwifery programs together with opportunities for community placements can have positive, long-term impacts on maternity service delivery and contribute to organisational change. While more evidence is required to determine whether a culturally respectful health workforce improves Aboriginal maternal and child health outcomes, enhanced access and increased satisfaction with services has been observed, especially where Midwifery Group Practice (MGP) models are adopted and Aboriginal staff participate in service delivery [29–31]. Noteworthy also is evidence of positive flow-on effects of MGPs to wider maternity services in terms of cultural responsiveness and early signs of improved health outcomes for Aboriginal women and their infants [30, 31].

Aboriginal women’s birthing stories highlight that in addition to empathy and relationship-building, cultural humility and “cultural champion” advocacy by midwives, a responsive, flexible health care system that accommodates cultural traditions around childbirth is required to prevent pregnant and birthing mothers feeling isolated and unsafe [7]. While an expanded Aboriginal maternity workforce is vital, culturally respectful midwives who advocate for change can also contribute to more positive birthing experiences for mothers and their families.

### Limitations

While the Indigenous unit was compulsory, participants who completed the remote clinical placement were selected based on interest and aptitude. The selection process likely influenced the high response rate in the initial and follow-up interviews (100% in both cases) and the value participants placed on the experience due to pre-existing receptivity. Recall bias is a potential limitation in longitudinal studies although this is mitigated to some extent if experiences recalled are powerful; many examples cited by participants fell into this category. Lastly, two of the nine midwives who completed the Indigenous unit and met the criterion for interview of this cohort were unavailable; missing data necessitates a more cautious interpretation of the findings.

## Conclusions

This study provides encouraging evidence that gains derived from well-designed and delivered Indigenous content in midwifery programs and opportunities for community placements can have a lasting and positive impact on maternity service provision to Aboriginal women. The privileging of Aboriginal voices both in the classroom and on the placement where students learned from community members, likely contributed to the success of both initiatives. Empathy and an enhanced capacity for relationship-building with Aboriginal women and their families were identified as important legacies of the unit and placement. Despite anxieties about causing offence, most participants were cognisant of culturally safe care and a number were active advocates for organisational change.

While organisational constraints are barriers to optimal culturally safe care, this study shows that the power of students’ transformative learning can endure in professional practice. Well-informed graduates have the capacity to become future change agents within health services that have not been exemplars of culturally safe care in the past. These findings can also inform scaled-up studies of Aboriginal women’s birthing stories where more evidence is required to determine the impact of culturally respectful care on women’s maternity experiences and health outcomes.

## Data Availability

Data sharing is not applicable to this article as no datasets were generated or analysed during the current study.

## Abbreviations

FBC: Family Birthing Centre
MGP: Midwifery Group Practice
NMSP: National Maternity Services Plan

## Footnotes

1 The Indigenous peoples of Australia include Aboriginal people and Torres Strait Islanders. In Western Australia, Torres Strait Islanders represent a very small proportion of Indigenous Australians and in this setting, there is a preference for the use of the term Aboriginal over terms such as Indigenous or abbreviations such as ATSI. For the purposes of this paper, we respectfully use the term Aboriginal and for simplicity use this term to refer to the Aboriginal and Torres Strait Islander peoples of Australia. The term Indigenous appears occasionally however, especially when used in quotations, units of teaching or reports. The terms are often used interchangeably and it is difficult to impose consistency.

2 ‘Birthing on Country’ refers to giving birth on the land of an Aboriginal woman’s own birth or on the land of the father of the child.

3 The Aboriginal and Torres Strait Islander Health Curriculum Framework was published in 2016 and hence did not guide implementation of the Indigenous unit which was introduced in 2012. Academics involved in the unit development, however, were subsequently part of a team awarded the contract to formulate the new curriculum framework.

4 ‘Women’s business’ refers to all aspects of reproduction, nurturing of children and ‘growing up’ strong Aboriginal women.

5 A Midwifery Group Practice model encourages women to see the same midwife through pregnancy, labour, birthing and during the post-natal period.

6 Noongar Boodjar refers to the ancestral country of the Noongar people in the south-west of Western Australia.
